# What Aspects of Religion and Spirituality Affect the Physical Health of Cancer Patients? A Systematic Review

**DOI:** 10.3390/healthcare10081447

**Published:** 2022-08-02

**Authors:** David Almaraz, Jesús Saiz, Florentino Moreno Martín, Iván Sánchez-Iglesias, Antonio J. Molina, Tamara L. Goldsby

**Affiliations:** 1 Department of Social, Work and Differential Psychology, Complutense University of Madrid, 28223 Madrid, Spain; dalmaraz@ucm.es (D.A.); jesus.saiz@psi.ucm.es (J.S.); fmoreno@psi.ucm.es (F.M.M.); 2 Department of Psychobiology & Behavioral Sciences Methods, Complutense University of Madrid, 28223 Madrid, Spain; i.sanchez@psi.ucm.es; 3 Department of Family Medicine and Public Health, University of California San Diego, La Jolla, CA 92093, USA; tgoldsby@health.ucsd.edu

**Keywords:** religion, spirituality, cancer, physical health, well-being

## Abstract

In recent years, the literature on the relationship between religion and spirituality (R/S) and the health of cancer patients has been flourishing. Although most studies focus on mental health, many study the physical health of these individuals. In order to summarize the findings of these studies, we reviewed the most recent research on this subject using the PubMed and PsycInfo databases. The objective of this systematic review was to recognize the primary R/S variables studied in research on physical health in cancer contexts. We found that spiritual well-being was the most-researched variable in studies of these characteristics, followed by R/S struggles and other variables such as religious coping; religious commitment or practice; or self-rated R/S. In general, R/S seems to have a positive association with the physical health of cancer patients, although the results are quite heterogeneous, and occasionally there are no relationships or the association is negative. Our results may assist in improving interventions that include spirituality in clinical settings as well as the development of holistic approaches, which may have a positive impact on the quality of life and well-being of cancer patients.

## 1. Introduction

Religion, the organized system of beliefs, practices, rituals, and symbols designed to facilitate closeness to the sacred or transcendent [1], has been part of human history since its inception. Spirituality may be considered intimately linked to religion, while also a unique and distinct concept. Koenig et al. [1], from a traditional point of view, define spirituality as the search for and discovery of the transcendent, as well as the relationship with the transcendent. Thus conceived, the two constructs are closely related. However, although conceptually, religious individuals may consider spirituality to be a central element of being religious, this need not be so from the point of view of predominantly spiritual people [2]. According to Schnell [2], spirituality may be conceptualized in two ways: a vertically transcendent spirituality that incorporates concepts of an eternal and supernatural God or higher power and a vertically transcendent spirituality that avoids reference to a supernatural reality but stresses the existence of an immanent absolute. It is noteworthy, therefore, that there are types of spirituality that do not require the existence of an organized system of beliefs or rituals. In this regard, Koenig et al. [1] highlight three additional ways of understanding spirituality apart from the traditional concept. The first of these may be considered a modern view of spirituality whereby spiritual people do not necessarily need to be religious and defined as “spiritual, but not religious”. A second manner of understanding spirituality would be a tautological view, which adds positive mental health and human values to the above. Finally, spirituality may be considered in terms of a modern clinical view, which would refer to it as another element that health care providers may consider in their approach to improving the well-being of patients. This can be seen, for example, in the relevance provided in certain health contexts, such as mental health, to religion and spirituality due to their significant implications in the prevalence, diagnosis, treatment, outcomes, and prevention of numerous disorders [3].

Koenig [4] argues that religion and spirituality (1) provide meaning and purpose during difficult life circumstances, which aid in psychological integration; (2) promote a positive view of the world that is optimistic and hopeful; (3) provide role models in the sacred writings that facilitate the acceptance of suffering; (4) give individuals a sense of indirect control over circumstances, reducing the need for personal control; and (5) offer a supportive community, both human and divine, to help reduce isolation and loneliness. Given these characteristics, religiosity/spirituality (R/S) may be considered a very useful tool to face difficult situations, such as an illness process. Thus, R/S is an aspect to be considered for health maintenance.

In recent years, religion and spirituality have begun to be studied in relation to health, with a substantial amount of literature now existing that documents the influence (or at least the association) of R/S on mental health [5,6,7,8], physical health [9,10], and even social health [11,12]. In general, R/S is not studied in a generic way. However, certain variables or dimensions are considered when describing this construct, such as attachment to God [13], trust in God [14], or spiritual well-being [15]. In fact, Hill and Pargament [16] propose that through these variables, which are conceptually more related to health, more can be discovered about the influence of religion and spirituality on physical and mental health. For this reason, numerous instruments have been developed to measure the different dimensions that make up R/S in health contexts [17].

As an aside, it is important to mention that this vast literature on religion, spirituality, and health is mainly composed of cross-sectional studies [18,19,20]. The difficulty of making causal inferences, the susceptibility to bias or the difficulty in interpreting the associations identified are some of the limitations that are typical of cross-sectional studies [21]. Therefore, it must be understood that many of the studies in this field have a series of problems that limit the possibility of making causal inferences. However, longitudinal studies, which are much more complex to carry out (especially in groups with serious diseases and therefore uncertain life expectancy), do allow for causal inferences.

Another limitation present in certain studies involves the small sample size. As stated by Hackshaw [22], studies with small samples may produce false-positive results or overestimate the magnitude of an association. These are problems that often appear in studies on religion, spirituality, and health, and that must be considered in our discussion regarding research in this field.

In any case, most of the research examining the relationship between R/S and health focuses on mental health, with a large number of studies showing the effects of R/S on this aspect of health [23,24,25]. As Koenig [26] discusses, it is to be expected that there are stronger relationships between mental health and R/S, since the latter involves psychosocial and behavioral aspects closer to mental health than to physical health.

However, as mentioned above, R/S has been studied in relation to physical health and has generally focused on the health of people with some type of physical pathology, such as respiratory disease [27], cardiovascular disease [28], or various types of chronic diseases [9,29]. Be that as it may, the relationships between R/S and physical health have been extensively documented [1,30,31,32].

It should also be made clear that since the constructs of physical and mental health have been shown to interact in numerous ways, it may be difficult to separate and identify the effective driving factors. Specific study designs, such as experimental RCTs or longitudinal epidemiologic design, may help in distinguishing the direction of effects. Authors such as Jones [33] have exposed this difficulty derived from the interaction between the physical and the mental. For this reason, numerous studies have been conducted involving mental health and physical health variables [34,35].

Nevertheless, part of this research has focused on the study of the relationship between R/S and physical health in cancer. In fact, although R/S may have general effects on health regardless of the disease, our study focuses on cancer, since focusing on this disease and its specific characteristics allows us to be more precise. In addition, oncologic disease is one of the leading causes of morbidity and mortality in the world [36] and results in significant physical, psychological, social, and spiritual impact on those who suffer from it. In general, the results obtained in these works are quite varied, usually as a result of the heterogeneity of the variables studied and the measures utilized, derived from the various existing conceptions of R/S [37,38].

In order to understand and summarize the heterogeneity of research results on the role of R/S in oncology patients, authors such as Jim et al. [39] conducted a series of meta-analyses that, again, appear to verify the diversity of results obtained, resulting, in some cases, contradictory results, as these findings go in different directions. These authors explored how the affective, cognitive, and behavioral aspects involving R/S affect the physical health of cancer patients. Generally, however, Jim et al. [39] found that R/S is associated with better patient-perceived physical health.

In view of this, we consider it relevant to inquire further into these results. Specifically, it is appropriate to examine the results found in research following the meta-analysis by Jim et al. [39] (which represents the latest review focused on the influence of R/S on cancer patients’ self-rated health), for several reasons.

In regard to the association of R/S and the physical health of oncology patients, research is necessary to understand the processes and mechanisms underlying this relationship [1,39,40]. If we can identify the different aspects of R/S that are associated with the physical health of these individuals and the psychosocial variables that may be involved in this association, we will be closer to identifying these mechanisms.

Moreover, through the identification of the variables included in these studies, it may be possible to determine potential moderating variables in these relationships [40], which may assist both in identifying mechanisms and explaining the manner in which relationships involved are strengthened or attenuated. This, in turn, may assist those who incorporate R/S as part of their therapeutic approach to guide their intervention. Likewise, following a more clinical view, identifying R/S variables and their impact on the health of cancer patients may assist in the development of treatment plans and/or holistic intervention in oncological contexts.

Therefore, in this study, we conducted a systematic review of the post-2015 literature with the aim of recognizing the main R/S variables examined by recent studies in relation to the physical health of cancer patients, as well as synthesizing the major results obtained in these studies. Thus, the question that guided our research was the following: how do various variables linked to religion and spirituality affect the physical health of cancer patients?

## 2. Materials and Methods

The quality standards of the PRISMA 2020 methodology [41] were followed to perform this systematic review. PRISMA 2020 is a reporting guide designed to address issues in the publication of systematic reviews, representing an important aid in the planning and conduct of these reviews by ensuring that all recommended information is captured [41]. The PRISMA 2020 checklist includes 27 items (some of which have sub-items) divided into seven sections or domains, related to each section of the review report. We used this guide for two reasons. First, this allowed the present authors to be cognizant of their own potential biases, thus minimizing the possible bias introduced in the conclusions and to guarantee the transparency of such a long and laborious process.

Second, the PRISMA 2020 guide was used as it represents an updated reporting guide that includes new recommendations and existing methodologies for conducting systematic reviews. Thus, it is a very complete tool that allows researchers to produce a transparent and accurate publication describing reasons the review was carried out, the procedure utilized by the researchers and what was discovered. After studying the PRISMA criteria and prior to the systematic review itself, a generic examination of the reviews prior to 2015 was performed in order to establish terminology and avoid duplication of concepts.

### 2.1. Search Strategy

A search was carried out in two specialized databases: PsycInfo and PubMed. The search occurred between February and March 2022, and included articles published between 2015 and March 2022.

The terms “religion”, “spirituality”, “cancer”, and “health” were used as search terms, linked to each other through the Boolean operator AND in order to objectify the search in any field, thus achieving a broader scope. Thus, the specific search string was “religion” AND “spirituality” AND “cancer” AND “health”.

### 2.2. Eligibility Criteria

Inclusion and exclusion criteria were established for the search.

The inclusion criteria were as follows:

1. Articles published between 2015 and March 2022.
2. Articles in English and Spanish.
3. Cancer population and survivors. Articles whose sample consisted of persons with cancer at the time of the study or cancer survivors were examined.
4. Individuals 18 years of age or older.
5. Empirical studies.
6. Studies that included measures of self-perceived or biomarker-derived physical health. Following Jim et al. [39], physical well-being, functional well-being, and self-assessed physical symptoms were utilized as measures of physical health. In addition, the presence/absence of biomarkers that provide an objective measure of the health status of individuals was added as a measure of physical health.

Based on these, the exclusion criteria were:

7. Population related to cancer but who were not patients or survivors: caregivers, family members, nurses.
8. Individuals under 18 years of age.
9. Review studies, theoretical articles, and case studies.
10. Studies whose only measure of R/S was religious and/or spiritual interventions. These types of studies were excluded as they did not apply to our objectives, since we were attempting to identify specific R/S variables that have an impact on physical health.
11. Studies that included only mental health measures.

### 2.3. Selection Process

First, a review protocol was drawn up that allowed us to have a structured plan of the entire process, thus attempting to avoid biases that may appear during the process, as well as ensuring the transparency of the review. This step specified the need for the study, the review question, the search strategy, the eligibility criteria, the study quality assessment system, the data extraction method, data synthesis and dissemination.

After performing the search based on the above strategy, duplicate articles were eliminated through the ZOTERO bibliographic manager. The titles and summaries of all the records obtained were read. A checklist was created in Microsoft Excel, where it was determined whether each inclusion criterion and exclusion criterion was met, as well as the relevance of the articles for our purpose.

Subsequently, the full papers pre-selected in the abstract reading phase were read and examined. Using the checklist created earlier, the inclusion and exclusion criteria were reapplied, selecting the articles to be included in the review.

### 2.4. Evaluation of the Methological Quality of the Articles

The quality of the selected research was analyzed using the McMaster University Critical Review Form for Quantitative Studies [42], although the criteria of Appelbaum et al. [43] and Levitt et al. [44] were checked as a complement. This evaluation tool was selected due to its suitability for various research designs. This form contains a total of 19 criteria, grouped into eight sections: purpose of the study, literature, sampling, evaluation of results, intervention, analysis of results, and conclusions/clinical implications. In this evaluation, it was decided to omit the three items referring to the intervention, since one of the exclusion criteria of this review was that the R/S variable used was only an R/S intervention. For this reason, a total of 16 criteria were used to evaluate methodological quality.

To be as precise as possible in this assessment, we adapted the scores of the original 19-criteria version for the 16 criteria used in our evaluation, establishing a proportionality relationship between the values. Thus, in our quality assessment, the score was as follows:

- Excellent: 16.
- Very good: 14 to 15.
- Good: 12 to 13.
- Acceptable: 10 to 11.
- Poor: 9 or less.

### 2.5. Extracted Data

In view of the inclusion and exclusion criteria, the research question and the objective of the study, the baseline information extracted and recorded from each article was as follows:

- Title, authors, year of publication and journal.
- Country in which the study was conducted.
- Study sample: number of participants, type and stage of cancer, and whether patient or survivor.
- Objective, research design and instruments used.
- Key variables: measures of R/S and physical health.
- Main results or findings of each investigation.

## 3. Results

### 3.1. Selected Articles

The PsycInfo search yielded a total of 101 articles, while PubMed yielded 341 results. After eliminating 62 studies that appeared to be duplications, 380 articles remained for the first reading of titles and abstracts. A total of 206 articles that met exclusion criteria or were irrelevant were discarded.

**Figure 1:**
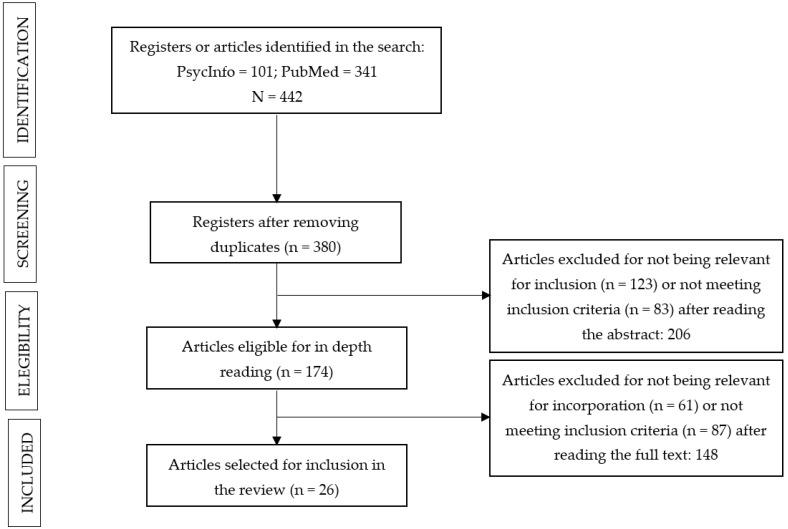
Article selection process.

The full text of the 174 articles was analyzed. After applying the inclusion and exclusion criteria, a total of 148 articles were obtained that were either ineligible or met exclusion criteria. Finally, a total of 26 articles were included in the review. The entire selection process may be viewed in Figure 1.

Although there was no inclusion criterion regarding the type of methodology used, the 26 articles included in the review employed a quantitative methodology. Of these, 22 used a cross-sectional design, while the remaining four were longitudinal studies.

The articles were published primarily in health care, public health, psychology, and oncology journals. There was not a substantial difference between the number of articles published in any given year. The year 2017 revealed the highest number of publications, a total of five. From 2018 to 2020, four articles were published per year, while in 2015 and 2021, three articles were published. In 2016, only two of the articles included in the review were published, while in 2022 (as of March) one article was published.

In regard to the countries in which the research studies were conducted, more than 50% were conducted in the United States (a total of 14). In addition, two of the studies were conducted in China. Of the remaining studies, one article was included in the review from each of the following countries: Saudi Arabia, Jordan, Iceland, Lebanon, India, Japan, Brazil, Chile, Turkey. Additionally, one article consisted of an international study involving several countries.

We also examined the various types of cancer that appeared in participants in the examined studies. Of these investigations, seven focused on a single type of cancer and one of the articles did not specify the type of cancer of the participants. The remaining articles did not focus their research on a particular type of cancer but included participants with disparate diagnoses. Not all of the articles consulted reported on all the types of cancer present in the sample; thus, the number of articles which each type of cancer mentioned appeared were analyzed.

Breast cancer appeared in the majority of articles (15), followed by genitourinary cancers (prostate, bladder, kidney, etc.), which appeared in 10 of the studies. Two other widely represented cancers were lung cancer and colorectal cancer, which appeared in nine and eight articles, respectively. Gynecological cancers (uterus, ovary, endometrium, fallopian tubes, etc.), hematological cancers (leukemia, lymphomas, myelomas, etc.) and gastrointestinal cancers (not including colorectal cancer) each appeared in seven investigations. The cancers appearing in a smaller number of studies were head and neck (four) and skin (two) cancers.

### 3.2. Quality Evaluation

Taking as a reference the criteria indicated by Law et al. [42], the methodological quality of all the articles evaluated ranged from good to very good, with the lowest score being 12 and the highest 15. Of the 26 articles selected, 21 were of very good quality (between 14 and 15), while the remaining 5 were of good quality (between 12 and 13). Table 1 displays the methodological quality of each article.

### 3.3. R/S Variables, Health Variables and Main Results

#### 3.3.1. R/S Variables

In two articles, the R/S variables used were simply religiosity and spirituality [45,46]. In those cases, these variables involved patients’ own self-perception of their R/S assessed through a single item. The remainder of the articles developed the R/S concept through various dimensions.

In 16 of the studies included in the review, the measure of religiosity/spirituality assessed was spiritual well-being. In 13 of these studies, spiritual well-being was understood as consisting of the three dimensions used by the FACIT-Sp12: meaning, peace, and faith [47,48,49,50,51,52,53,54,55,56,57,58,59]. In two other studies, spiritual well-being was understood as consisting of other dimensions, specifically those that comprise the EORTC QLQ-SWB32: existential, relationship with self, relationship with others, and relationship with someone/something greater [60,61]. The last article using spiritual well-being as a measure of R/S studied this variable (spiritual well-being) in regard to relationship with self and others, religious/spiritual beliefs and practices, and existential [62].

The next most-frequently used R/S variable in the research was religious/spiritual struggles, which is examined in three of the articles [63,64,65].

However, in other cases, R/S is assessed through variables such as spiritual experiences, religious beliefs and practices, congregational support, or religious coping [66,67,68,69]. The latter represents an R/S variable present in the previous three articles mentioned. Finally, one of the articles used spiritual pain as an R/S variable [70].

#### 3.3.2. Physical Health Variables

Regarding the physical health variables assessed in the research, we found that the variables that appeared most frequently pertain to physical symptoms, such as pain or fatigue, in a total of 16 articles [46,49,50,52,55,56,60,62,63,64,65,67,68,69,70]. Symptoms are followed by physical and functional well-being, which appeared as a health variable in a total of ten articles [47,48,50,51,53,55,56,57,58,59]). Physical quality of life appeared in two studies as a health variable [45,70]. In addition, physical functioning was examined as a physical health variable in five articles [52,60,61,62,67]. Finally, one article specifically used physical health as the main variable [54].

While all of the above physical health variables were subjective in nature, as they were self-reported measures, it is noteworthy that two of the articles used biomarkers such as cortisol [67] or telomere length [66], which are objective physical health measures.

#### 3.3.3. Main Research Results

The R/S variables mentioned above showed a varied association with the different health variables. Firstly, generic R/S, which does not specify any dimension, was associated with lower levels of pain in a cross-sectional study [46]. In another cross-sectional study, Al Ahwal et al. [66] found no significant associations between R/S and telomere length, a biomarker representing an objective measure of physical health status. In addition, R/S was found to be associated with increased physical well-being longitudinally [45].

As already mentioned, spiritual well-being is the R/S variable that appeared most frequently in the articles included in the review. Likewise, it was observed that it appeared in various ways, depending upon the dimensions that the authors established as components of spiritual well-being.

Spiritual well-being, understood as a construct composed of meaning, peace, and faith, was related in various ways to the physical and functional well-being of oncology patients, offering similar results in some cases and disparate results in others. In several cross-sectional studies, all three dimensions were positively and significantly associated with physical well-being and functional well-being [47,50,55]. In fact, some studies also found significant associations between total spiritual well-being and physical and functional well-being [55,59]. In contrast, there were studies that found positive associations of these dimensions only with functional well-being [48,51], and others that did so with physical well-being [57].

The results from the various studies varied depending on the dimension analyzed. Cheng et al. [53] observed that all three dimensions were positively associated with functional well-being, although meaning was not associated with physical well-being and faith was, albeit in a negative direction. Several studies found that while meaning and peace were associated with both types of well-being, faith was associated only with functional well-being [58,59]. The latter studies had a cross-sectional design. In contrast, through a longitudinal study, Leeson et al. [56] found that meaning/peace predicted greater physical and functional well-being.

One longitudinal study examined spiritual well-being but focused on the physical health status of individuals with cancer, measured through the physical component of the SF-36, as a self-perceived physical health variable [54]. The authors of this study found that meaning and peace were longitudinally positively and significantly associated with health status, while faith was not associated with this variable.

Spiritual well-being (composed of meaning, peace, and faith) was also related to physical symptoms in cancer patients, with similar heterogeneous results. Meaning and peace were found to be negatively correlated with various symptoms (fatigue, pain, dyspnea, loss of appetite, etc.) according to different cross-sectional investigations [49,52,55]. Only Brown et al. [50] found no relationships between meaning and peace and physical symptoms. In contrast, faith varied in its influence on symptoms. In some cases, higher faith was found to be associated with a higher level of physical symptoms [49], while in other cases, the association was negative [52]. This is similar to the longitudinal study by Leeson et al. [56], which found that meaning/peace predicted less fatigue and pain over time, whereas faith was associated only with greater fatigue.

The purported influence of spiritual well-being on the physical health of individuals with cancer was also analyzed in several cross-sectional studies that conceived of spiritual well-being as composed of relational (with self, others, or someone or something higher) or existential dimensions [60,61,62]. In this regard, two of the studies observed no relationship between global spiritual well-being with functional and physical symptoms [61,62], while Chen et al. [60] did observe significant relationships between these variables. The various dimensions correlated positively with physical functioning and negatively with physical symptoms, with the exception of the dimension of relationship with something or someone higher, which was not associated (or in the case of physical functioning, was negatively associated).

Another major variable, R/S struggles, was studied cross-sectionally in relation to physical symptoms. Two studies in our review found no significant associations between R/S struggles and pain [64,65]. In contrast, Damen et al. [63] did observe a positive association between symptomatic burden and R/S struggles. A single study, conducted by Pérez-Cruz et al. [70], evaluated spiritual pain, understood as “a deep pain in your soul/being that is not physical” (p. 2), observing that it was associated with lower physical quality of life and a higher number of symptoms.

Finally, a few studies focused on behavioral R/S variables. Among them, R/S coping stood out. On the one hand, several cross-sectional studies observed associations between negative coping and greater physical discomfort, considered as bodily discomfort related to physical weakness and disability [68], as well as worse physical functioning and more pain [67]. On the other hand, other behavioral aspects such as positive spiritual experiences and private religious practice were associated with better physical functioning and lower pain and, in addition, the former were also associated with lower cortisol levels [67]. In addition, a longitudinal study found that negative coping led to more sleep problems, as well as finding that private religious engagement was associated with more cancer-related symptoms [69].

Table A1, included in Appendix A, provides a detailed description of each study, linking the objectives, measures, and results with reference to the type of design and the main characteristics of the sample.

## 4. Discussion

This study aimed to detect the primary religious and spiritual variables considered in recent research in relation to the physical health of oncology patients, as well as to summarize the main findings of such research.

We have observed that recent research in this field particularly focuses on spiritual well-being. Spiritual well-being is often described as “a dynamic and affective dimension of religion and spirituality that impacts the way that people experience, understand and live their lives” [71]. In this sense, Leung and Pong [72] state that this well-being is an indicator of spiritual health, understood as a condition that guides individuals to identify their meaning and purpose in life, based on connections with others and the transcendent. Thus, spiritual well-being is beginning to be considered as another element of people’s holistic health, along with physical and mental health [72,73]. We believe this represents one reason why the use of spiritual well-being as a measure of R/S in health contexts has been increasing in recent years. In addition, it is worth noting that important previous studies have already highlighted the reasons why it is appropriate to use spiritual well-being as a measure of R/S in oncology settings. For example, Peterman et al. [74] argue that spiritual well-being allows for the assessment of spirituality across a broad spectrum of religious traditions and for people who consider themselves spiritual but not religious. Other authors, such as Puchalski [75], express this importance by referring again to spiritual well-being as a determinant of the integral health of these patients and, therefore, of their quality of life.

Spiritual well-being is generally conceived as a multidimensional construct which includes meaning, peace, and faith [76]. Thus understood, in the articles included in the review, this well-being has been related to various physical health variables in a number of ways. It is observed that, in general, greater spiritual well-being is associated with better physical health outcomes in oncology patients. In any case, considering these dimensions of spiritual well-being, it should be noted that the results obtained for the faith variable oblige us to be cautious when drawing conclusions. In addition to being heterogeneous, in many cases they may be contradictory, as associations are found in different directions or, on occasion, no relationship is found between faith and the physical health of cancer patients. It is possible that the general difference between the results of meaning/peace and faith pertains to their conceptualization in the FACIT-Sp, the main instrument for measuring these constructs. As discussed in one of the early and major studies on this topic, one of the strengths of the FACIT-Sp is that the Faith subscale can measure a dimension of spirituality that overlaps with or is reinforced by religion, whereas the Meaning/Peace subscale measures a dimension that is more independent [74]. Thus, it seems that the results linked to faith may be contradictory due to their association with religion. In this sense, although religion in many cases has positive effects on health, it may at times have a detrimental influence on it, as observed in one of the longitudinal articles included in the review [56]. Another possible reason is found in the research of Saiz et al. [77]: simple membership in a religious doctrine, regardless of spirituality, is not a reliable predictor of health-related benefits. In other words, faith without a daily practice and spiritual life may have counterproductive effects on health [77].

Spiritual well-being has also been defined as a construct composed of relational (with self, with others, with something or someone higher) or existential dimensions [60,61,62]. In general, spiritual well-being as measured cross-sectionally by these dimensions has been found to be positively associated with physical health of cancer patients, with certain exceptions concerning the dimension relationship with someone or something higher, which is not associated with physical health and, on one occasion, is negatively associated with physical functioning.

Thus, it may be important for research to attempt to unify the concept of spiritual well-being in order to understand how it influences or is associated with people’s physical health. As already mentioned, mental and physical health are closely interconnected, although they are generally conceived as different aspects; together with these, spiritual well-being appears as another element of health that must be considered. In this sense, considering the “mental” as a function of the physical organism may be helpful in explaining the relationship between spiritual well-being and physical health. In this regard, the research by Sleight et al. [57] included in this review sheds light on how spiritual well-being can attenuate the effect that some mental health problems have on physical health. Other included articles, such as the one by Kamijo and Miyamura [55], show how spiritual well-being is associated not only with physical and functional well-being, but also with mental and social well-being. Thus, following the previous line of reasoning, we could assume that spiritual well-being may be related to physical health through the influence of various aspects related to psychosocial well-being. Current research on religion, spirituality, and physical health therefore needs to focus on the study of psychosocial determinants (e.g., psychological well-being and social well-being) that influence these relationships, in order to understand the mechanisms by which R/S impacts physical health. However, while more research of this type is needed, there is the possibility that R/S acts on people’s health through as yet unknown pathways, which could include some form of divine intervention. This may be an option that researchers in any field should keep open. In any case, these avenues of research would represent a breakthrough in treating not only physical, but also holistic, health.

In addition, it seems necessary to emphasize those dimensions that imply a relationship with the transcendent. In the case of the faith dimension of the FACIT-Sp12 and the relationship with someone or something higher dimension of the EORTC QLQ-SWB32, it is observed that at times the results are either not significant or go in the opposite direction of better physical health. Both dimensions refer to the individual’s belief in God (i.e., it may have a religious connotation) or to spiritual beliefs. That is, they would belong to what Jim et al. [39] call the cognitive dimension of R/S. In this regard, the findings of Jim et al. [39] are congruent with the present review, as they do not observe a significant relationship between R/S beliefs and physical health in individuals with cancer.

Another variable that has been related to physical health in the review (specifically physical symptoms), specifically in a cross-sectional manner, is R/S struggles. In general, either no relationship was observed between the two variables, or a higher level of R/S struggles was found to be associated with a higher symptom burden. As with R/S struggles, there are other religious/spiritual variables that also involve a confrontation with the divine or with one’s own practices and beliefs, such as spiritual pain, which is also associated with poorer physical health in oncology patients [70]. In view of this possible relationship between R/S struggles or spiritual pain and physical health, the need to attend to and care for the spiritual needs of oncology patients becomes evident. In fact, several studies have highlighted the importance of spiritual care as part of the holistic health care of individuals [78,79].

In general, the important role of R/S in the psychosocial adjustment of individuals to cancer diagnosis and treatment has been highlighted, with a view to improving physical and mental well-being [80], through aspects such as religious coping. Nevertheless, we believe that what was observed in several of the articles in this review brings to light the need to address the negative R/S aspects present in oncology patients, such as R/S struggles or spiritual pain, within clinical and health care settings. This idea is related to the negative association that certain R/S variables have with physical health, mentioned above. Likewise, much research has been devoted to analyzing the relationships between variables of this type, such as R/S struggles [81,82] or mistrust in God [83] in relation to mental health. Therefore, we consider it necessary to further explore the effect on the physical health of oncology patients of R/S variables that imply a conflicting relationship with the divine or spiritual or a doubt about one’s own beliefs.

Be that as it may, these last-mentioned variables, together with others such as meaning or peace, would compose the affective dimension of R/S proposed by Jim et al. [39]. Thus, as with these authors, our review shows that this type of variable is generally associated with physical health.

Finally, several studies focus on a more behavioral dimension of R/S and its relationship with cancer, as Jim et al. [39] would term it, which likewise present heterogeneous results. Among these aspects we can highlight R/S coping, religious engagement, spiritual experiences, or religious practices [67,68,69]. In any case, these behavioral aspects of R/S are to a certain extent contradictory to the synthesis by Jim et al. [39], who did not observe an overall significant relationship between aspects such as coping or religious practice and physical health. However, for example, one of the longitudinal studies [69] has shown that certain behavioral aspects (such as negative coping or private religious engagement) have a negative impact on the physical health of oncology patients.

It is worth noting that, although R/S is generally studied in relation to perceived physical health, the cross-sectional study by Hulett et al. [67] represents one of the few attempts to test how R/S is associated with physical health objectively, through biomarkers that reflect the correct or incorrect functioning of the organism, such as cortisol itself. In the case of studies on the relationship between R/S and biomarkers, it also happens that a significant number of them are focused on biomarkers related to mental health, such as Posterior EEG Alpha (a putative biomarker of clinical outcome in major depression) [84]. Thus, and in view of the effects that R/S has on various aspects of physical health, more research is needed to understand the relationships between R/S and neuroendocrine and immune functions as represented by biomarkers to the extent that these, as proposed by Koenig et al. [1], affect susceptibility to, and recovery from, disease. Research of this type may be the way forward to definitively understand how the relationships between R/S and individuals’ health behave, although it requires a complex methodology that makes it difficult to carry out in many cases.

This need is evident in another of the studies in this review, which focuses on a particular aspect of R/S, attempting to associate it with telomere length, another biomarker representing an objective measure of physical health status [66]. Furthermore, this non-specific and general R/S has been associated with better physical health both longitudinally [45] and cross-sectionally [46]. Similarly, Jim et al. [39] observed that R/S measured in a general way (i.e., through single items that properly measure religiosity and/or spirituality), without focusing on affective, cognitive, or behavioral aspects, is associated with better physical health in oncology patients.

### Limitations and Future Implications

In any case, this review of the literature on the relationships between R/S and physical health in individuals with cancer has a number of limitations.

The main limitation involves the cross-sectional design used by the majority of studies on religion, spirituality, and physical health. This is reflected, therefore, in the fact that 22 of the 26 articles that have been included in the review present this type of design. As noted above, the problem with cross-sectional studies is that they do not allow us to infer causality, but only whether or not there is an association between variables. In this sense, cross-sectional data obtained in these types of studies may be misinterpreted as causal inferences (when they should be interpreted in terms of association), which leads to further increase in the heterogeneity of the results. For this reason, it is worth highlighting the need for new studies that address the relationships between religion, spirituality, and health through longitudinal or other RCT-like epidemiological designs. However, in the case of individuals with certain diseases, it is even more complicated to perform long-term studies. To mitigate the difficulty of this type of study in populations with a real risk of death, the time periods for follow-up of the measure could be limited and less invasive forms of data collection than those usually used could be established. In this regard, a protocol for a cohort study involving cancer patients in a longitudinal setting to analyze the relationship between R/S and health in this population has recently been published [85], which could be considered as a reference for a design that can reduce the shortcomings of cross-sectional designs. Nevertheless, despite the weaknesses of the cross-sectional designs discussed by Wang and Cheng [21] and mentioned in the introduction, all articles that met the defined inclusion criteria were included in the sample.

In addition, it is difficult to synthesize the results of studies with these characteristics. The variety of measures used is an obstacle to the use of other methods such as meta-analysis. In fact, the authors are generally transparent when defining the constructs that they deal with in their articles, thus making it possible to observe the disparity (or in some cases, the similarity) between one and the other. In many cases, the same variable is measured in different ways and through a variety of dimensions. A very clear case is that of spiritual well-being, which is at certain times understood as composed of meaning, peace, and faith, and at other times as consisting of the relationship with self, the relationship with others, or the relationship with someone or something higher and existential aspects. It occurs in a similar manner, for example, with R/S struggles. At times, these are understood as a form of negative coping, while at other times they involve a number of dimensions derived from the focus of conflict, such as struggles with the divine; struggles with the demonic; interpersonal struggles; moral struggles; struggles with doubts; or struggles with ultimate meaning. Therefore, we believe that a consensus is needed to conceptualize certain aspects of R/S closely related to health as proposed by Koenig [37], which may assist in improving holistic interventions that consider the religiosity and spirituality of oncology patients. In addition, this consensus would allow the distinction between affective, cognitive, and behavioral aspects of R/S proposed in meta-analytic reviews such as Jim et al. [39], which may assist in the replicability of this type of studies.

Finally, we would like to highlight sample and cultural diversity as a limitation that may affect the heterogeneity of results. Although most of the studies have been conducted in the United States, a large number of the studies have been carried out in very diverse cultural contexts and have used very different and on occasion too small sample sizes. More studies are needed that allow us to test for differences between cultures, as well as using similar large samples to facilitate generalization of the findings.

In any case, through this review we have been able to recognize the main religious and spiritual variables that are associated (in various ways) with different measures of physical health in cancer patients. We consider that the identification of these types of variables represents a step forward in the understanding of the relationships between R/S and the health of individuals with cancer. As we previously proposed, if we know which elements of R/S affect physical health and how these elements are related to other psychosocial aspects that present during the oncological process, we will have an increased likelihood of understanding the mechanisms by which R/S impacts the physical health of these patients.

## 5. Conclusions

This review summarizes the results of research on the relationships between R/S and physical health of cancer patients in recent years. It has been observed that the various R/S variables (spiritual well-being, R/S struggles, religious coping or self-rated R/S, among others) examined in the review generally displayed a positive association with the physical health of these patients. However, occasionally, R/S may be negatively associated with or unrelated to health. Our findings may assist in the treatment of various aspects of religiosity and spirituality, whether positive or negative, in health care contexts in order to improve the well-being and quality of life of individuals affected by cancer.

## Figures and Tables

**Table 1:** Methodological quality of the articles included in the review.

Authors	Total (%)	Methodological Quality
Al Ahwal et al., 2018	14/16 (87.5%)	Very good
Al-Natour et al., 2017	15/16 (93.75%)	Very good
Asgeirsdottir et al., 2017	12/16 (75%)	Good
Bai et al., 2016	13/16 (81.25%)	Good
Best et al., 2015	15/16 (93.75%)	Very good
Brown et al., 2015	14/16 (87.5%)	Very good
Canada et al., 2016	15/16 (93.75%)	Very good
Cannon et al., 2022	15/16 (93.75%)	Very good
Chaar et al., 2018	14/16 (87.5%)	Very good
Chen et al., 2021	15/16 (93.75%)	Very good
Cheng et al., 2019	15/16 (93.75%)	Very good
Damen et al., 2021	14/16 (87.5%)	Very good
Gielen et al., 2017	12/16 (75%)	Good
Goyal et al., 2019	15/16 (93.75%)	Very good
Hulett et al., 2018	15/16 (93.75%)	Very good
Kamijo and Miyamura 2020	15/16 (93.75%)	Very good
King et al., 2017	15/16 (93.75%)	Very good
King et al., 2018	15/16 (93.75%)	Very good
Leeson et al., 2015	15/16 (93.75%)	Very good
Mendoça et al., 2020	15/16 (93.75%)	Very good
Narayanan et al., 2020	15/16 (93.75%)	Very good
Pérez-Cruz et al., 2019	12/16 (75%)	Good
Rohde et al., 2019	14/16 (87.5%)	Very good
Sleight et al., 2021	14/16 (87.5%)	Very good
Walker et al., 2017	13/16 (81.25%)	Good
Yilmaz and Cengiz 2020	14/16 (87.5%)	Very good

## Data Availability

The study did not report any data.

## Appendix A

**Table A1:** Description of studies included in the systematic review.

Author (Year)	Objectives	Sample	Measures	Design	Results
Al Ahwal et al., 2018	(1) To explore the relationship between telomere length and religious participation; (2) To examine the demographic, social, psychological, and cancer-related correlates and mediators of the relationship	n = 50; 41–64 years; 52% female. Saudi Arabia	Sociodemographic data; 13-item Muslim Religiosity Scale; 11-item version of the Duke Social Support Index; 17-item Hamilton Depression Rating Scale; DSM-IV criteria checklist of mental disorders; Personal and family psychiatric history; Depression history; Family history of mental and nervous disease; Blood test telomeres	Cross-sectional	Positive association between religiosity and telomere length, weak and not significant. Neither religious beliefs nor religious practices were significantly associated with telomere length
Al-Natour et al., 2017	(1) To investigate the relationship between spirituality and dimensions of quality of life for Jordanian women diagnosed with breast cancer, including physical, social, emotional and functional well-being	n = 150; 25–69 years; 100% female. Jordan.	Sociodemographic data; Arabic version of FACIT-Sp	Cross-sectional	Positive and significant association between spiritual well-being and physical and functional well-being
Asgeirsdottir et al., 2017	(1) To examine the feasibility of the Icelandic version of the provisional EORTC SWB measure; (2) To explore the relationship between spiritual well-being and quality of life of palliative care patients in Iceland	n = 30; 51–83 years; 73.3% female. Iceland.	Provisional EORTC QLQ-SWB; EORTC QLQ-15-PAL	Cross-sectional	Global health/quality of life status correlated moderately and positively with total spiritual well-being. Physical functioning and physical symptom scale did not correlate with spiritual well-being
Bai et al., 2016	(1) To examine individual patterns of spiritual well-being in newly diagnosed patients with advanced cancer using cluster analysis	n = 52; 21 years or older; 46.2% female. USA.	FACIT-Sp12; FACT-G; PHQ-9; Self-rated health item; Self-rated spirituality item	Cross-sectional	There were significant differences in functional well-being as a function of meaning, peace and faith, but no such differences were observed for physical well-being. The cluster with the highest meaning, peace and faith, had the highest level of functional well-being
Best et al., 2015	(1) To examine the extent to which spirituality is associated with self-rated health among African American cancer survivors compared to other racial/ethnic groups; (2) To examine the extent to which cancer-related problems mediate the relationship between spirituality and self-rated health	n = 9006; 23–100 years; 55% female. USA.	Sociodemographic and health data; Self-rated health item; Cancer Problems in Living Scale; FACIT-Sp12	Cross-sectional	Meaning was negatively and significantly associated with physical symptoms in both groups. There were significant negative associations between peace and physical symptoms in both groups, although these associations were stronger in African Americans. Faith was positively and significantly associated in both groups
Brown et al., 2015	(1) To assess whether an individual’s level of meaning/peace predicts various measures of quality of life and mental well-being; (2) To identify goals that can improve the overall spiritual well-being and quality of life of ovarian cancer patients	n = 104; 33–83 years; 100% female. USA.	Sociodemographic data; FACIT-Sp12; FACT-O; HHI; ESAS; Death Anxiety Scale; HADS; Coping dimension of the BMMRS	Cross-sectional	Greater meaning/peace predicted higher levels of physical and functional well-being and fewer sleep problems. No correlations were found between meaning/peace and symptoms more physical in nature (pain, fatigue, etc.)
Canada et al., 2016	(1) To clarify the relationship between dimensions of spiritual well-being and quality of life in cancer survivors	n = 8405; 23–100 years; 55.1% female. USA.	Sociodemographic data; FACIT-Sp12; Physical component of the SF-36; Mental component of the SF-36	Cross-sectional	Meaning, peace and faith were associated with the physical component of the SF-36 (functional quality of life)
Cannon et al., 2022	(1) To explore the relationship of spirituality and religiosity as it relates to the physical and mental quality of life of cancer survivors	n = 551; 19–85 years; 36.5% female. USA.	Sociodemographic data; FACIT-Sp; Two items on religiosity (beliefs and practices); Short-Form-12 Health Survey	Longitudinal	No significant interaction was observed between religiosity, spirituality and quality of life over time. Physical well-being of cancer survivors with low spirituality and religiosity was significantly lower that those with high religiosity and spirituality. The effect of spirituality on physical quality of life was only significant among highly religious participants
Chaar et al., 2018	(1) To assess the impact of spirituality on quality of life, depression and anxiety in Lebanese cancer patients	n = 115; 18 years or older; 67% female. Lebanon.	Sociodemographic and clinical data; Arabic version of EORTC-QLQ-C30; Arabic version of FACIT-Sp12; Arabic version of HADS	Cross-sectional	Meaning was uniquely associated with dyspnea, in a negative and significative manner. Peace was negatively and significantly associated with fatigue, pain, dyspnea and loss of appetite. Faith was negatively and significantly associated with dyspnea and loss of appetite. All dimensions and total spiritual well-being were positively associated with overall health and quality of life status and role functioning
Chen et al., 2021	(1) Investigated spiritual well-being and its association with quality of life, anxiety and depression in patients with gynecological cancer	n = 705; 18 years or older; 100% female. China.	EORTC QLQ-SWB32; EORTC QLQ-C30; HADS	Cross-sectional	Total spiritual well-being and the existential, relationship with self, and relationship with others correlated significantly and positively with physical functioning and negatively with most physical symptoms. The relationship with something superior dimension correlated only positively and significantly with loss of appetite
Cheng et al., 2019	(1) To explore factors associated with spiritual well-being among cancer patients and the relationship between spiritual well-being and quality of life	n = 185; 18 years or older; 53% female. China.	Sociodemographic and clinical data; FACT-G; FACIT-Sp12	Cross-sectional	All dimensions were significantly and positively related to functional well-being. In addition, peace was positively associated with physical well-being, while faith was negatively associated with physical well-being. Peace and faith had a predictive power on physical well-being, and these together with meaning predicted an important part of functional well-being
Damen et al., 2021	(1) To examine the prevalence, predictors and correlates of R/S struggles in the palliative care cancer population	n = 331; 55–93 years; 56% female. USA.	Sociodemographic data; RRS-14; Three items on religious characteristic; ESAS; PDI; QUAL-E	Cross-sectional	Higher symptom burden was significantly and positively associated with higher R/S struggles. Higher scores in all subdomains of R/S struggles were associated with higher symptom burden, except for interpersonal struggle
Gielen et al., 2017	(1) To examine the prevalence and nature of spiritual distress in Indian palliative care patients	n = 300; doesn’t specify age range; 49.3% female. India.	Sociodemographic data; Newly developed questionnaire for the study of spirituality in Indian palliative care patients; Item to assess pain	Cross-sectional	Statistic significant differences in pain scores were observed between the different clusters. Spiritually distressed patients suffered more severe pain, while patients who trust in God suffered less
Goyal et al., 2019	(1) To examine the reciprocal relationship between spirituality and physical health status in breast cancer survivors	n = 634; 18 years or older; 100% female. USA.	Sociodemographic and cancer-related data; FACIT-Sp12; Physical component of the SF-36	Longitudinal	Meaning and peace correlated significantly with the physical component both cross-sectionally and longitudinally. The correlations between faith and the physical component were very low and not significant
Hulett et al., 2018	(1) To determine the feasibility and acceptability of a salivary cortisol self-collection protocol; (2) To examine the relationships between R/S, health perceptions and daytime salivary cortisol	n = 41; 51–88 years; 100% female. USA.	Sociodemographic data; BMMRS; SF-36v2; Cortisol	Cross-sectional	Positive spiritual experiences were the only spiritual variable that demonstrated a statistically significant relationship with peak cortisol. In general, poorer physical health was inversely associated with positive spiritual experiences and private religious practices. Specifically, positive religious experiences, spiritual coping and private religious practices were inversely and significantly correlated with physical function and bodily pain. Negative congregational support was significantly and negatively associated with physical function. Forgiveness, positive congregational support, and negative spiritual experiences were not significantly associated with physical health
Kamijo y Miyamura 2020	(1) To examine patients’ level of spirituality, the relationship between spirituality and physical pain, and the association between spirituality and quality of life among patients undergoing chemotherapy for cancer	n = 176; 22–88 years; 75% female. Japan.	Sociodemographic and clinical data; OPTIM Screening Sheet; VAS; FACIT-Sp12; FACT-G	Cross-sectional	Total spiritual well-being and all its dimensions correlated significantly with physical and functional well-being. In addition, faith and meaning/peace correlated significantly with loss of appetite, and the latter dimension also correlated significantly with insomnia.
King et al., 2017	(1) To describe the prevalence, demographic and medical correlates, and emotional and quality of life correlates of R/S struggles in HCT survivors	n = 1449; 18–89 years; 49% female. USA.	Sociodemographic and medical data; Brief RCOPE negative coping subscale; General health and pain subscales of the SF-36; Existential and social support subscales of the McGill Quality of Life Questionnaire; PHQ-8; Question about whether they have graft-versus-host disease	Cross-sectional	R/S struggles were not significantly associated with graft-versus-host disease or with pain or general health
King et al., 2018	(1) To study the relationship between R/S struggle and existential quality of life; (2) To examine the demographic, medical, emotional and social correlates of quality (of life?) in young people	n = 172; 18–39 years; 55.8% female. USA.	This article presents the same measures as the previous one (see King et al., 2017)	Cross-sectional	R/S struggles were not significantly associated with graft-versus-host disease or with pain or general health
Leeson et al., 2015	(1) To investigate changes in spirituality among HCT recipients over time; (2) To assess the extent to which spirituality before HCT predicted important dimensions of quality of life after transplantation	n = 220; 19–74 years; 38.2% female. USA.	FACIT-Sp12; IDAS; FSI; BPI; Physical and functional well-being dimensions of the FACIT	Longitudinal	Meaning/peace significantly predicted less fatigue and pain, as well as greater physical and functional well-being during the 12 months after transplantation. Faith was associated only with increased fatigue. Meaning/peace before HCT predicted changes in fatigue and physical well-being over time.
Mendoça et al., 2020	(1) To analyze the relationships between the subjective experience of distress and the use of R/S coping in adult patients receiving chemotherapy	n = 100; 18 years or older; 47% female. Brazil.	Sociodemographic data; ISDEI; ECOG Scale; VAS; RSC-Brief	Cross-sectional	Negative coping correlated weakly, significantly and positively with physical distress
Narayanan et al., 2020	(1) To identify the frequencies of spontaneously written religious content in participants’ emotional writing samples and the extent to which the religious content of their writing was associated with a validated self-report R/S scale and cancer-related psychosocial symptoms and outcomes	n = 138; 18 years or older; 40% female. USA.	Sociodemographic data; Religious content of the writings; Ironson-Woods Spirituality/Religious Index; BSI; BFI; PSQI; MOS-SSS	Longitudinal	Private religious engagement was negatively associated with fatigue. Negative religious coping was positively and significantly associated with sleep problems. Private religious engagement was significantly and negatively associated with cancer-related symptoms throughout the follow-up period, whereas no other R/S variable was associated with symptoms in that period
Pérez-Cruz et al., 2019	(1) To characterize the association between spiritual pain and quality of life in a group of advanced cancer patients in a palliative care clinic	n = 208; 18 years or older; 50% female. Chile.	Sociodemographic data; MDAS; ESAS; ESAS-F; HADS; EORTC-QLQ-C15-PAL; Self-rated spirituality and religiosity items	Cross-sectional	Spiritual pain was associated with poorer overall quality of life and poorer physical quality of life. It was also associated with fatigue, drowsiness, anorexia, dyspnea, sleep problems and general physical symptom burden
Rohde et al., 2019	(1) To report on an additional multivariate analysis, to investigate the relationships between sex, age and spiritual well-being of patients receiving palliative care	n = 451; 18–89 years; 54% female. International.	QLQ-SWB32; QLQ-C14-PAL	Cross-sectional	Spiritual well-being correlated significantly and positively with overall quality of life. No correlation was observed between spiritual well-being and physical functioning. A significative negative relationship of spiritual well-being with insomnia, fatigue and constipation was observed. All dimensions, with the exception of relationship with others, were associated with physical functioning and with one or more physical symptoms
Sleight et al., 2021	(1) To assess the extent to which spiritual well-being moderates the relationship between anxiety and physical well-being in a diverse, community-based cohort of newly diagnosed cancer survivors	n = 5506; 21–84 years; 60% female. USA.	Meaning/peace subscale of the FACIT-Sp12; PROMIS Anxiety short form; FACT-G	Cross-sectional	A positive direct effect of meaning/peace on physical well-being was observed. A significant interaction was found between meaning/peace and anxiety, indicating that spiritual well-being moderated the relationship between anxiety and physical well-being.
Walker et al., 2017	(1) To investigate the relationship between psychological state and traits in self-reported religious beliefs	n = 43; 52–79 years; 100% male. USA.	FACT-P; FACIT-Sp-Ex; PHQ-9; NEO Five-Factor Inventory	Cross-sectional	Meaning/peace correlated significantly and positively with physical well-being, functional well-being, and well-being related to prostate health. Faith was significantly associated with functional well-being, but not with physical well-being
Yilmaz y Cengiz 2020	(1) To assess the relationship between spiritual well-being and quality of life in cancer survivors	n = 150; 20–65 years; 61.3% female. Turkey.	Sociodemographic data; FACIT-Sp12; FACT-G	Cross-sectional	Significant positive correlations were found between total spiritual well-being and its dimensions with physical and functional well-being, with the exception of faith, which was only associated with functional well-being

## References

[1] Koenig H.G., King D.E., Carson V.B. (2012). Handbook of Religion and Health.

[2] Schnell T. (2012). Spirituality with and without religion—Differential relationships with personality. Arch. Psychol. Relig..

[3] Moreira-Almeida A., Sharma A., van Rensburg B.J., Verhagen P.J., Cook C.C.H. (2016). WPA Position Statement on Spirituality and Religion in Psychiatry. World Psychiatry.

[4] Koenig H.G. (2009). Research on religion, spirituality, and mental health: A review. Can. J. Psychiatry.

[5] Weber S.R., Pargament K.I. (2014). The role of religion and spirituality in mental health. Curr. Opin. Psychiatry.

[6] Holt C.L., Roth D.L., Huang J., Clark E.M. (2018). Role of Religious Social Support in Longitudinal Relationships between Religiosity and Health-Related Outcomes in African Americans. J. Behav. Med..

[7] Vitorino L.M., Lucchetti G., Leão F.C., Vallada H., Peres M.F.P. (2018). The association between spirituality and religiousness and mental health. Sci. Rep..

[8] Saiz J., Chen-Chen X., Mills P.J. (2021). Religiosity and Spirituality in the Stages of Recovery from Persistent Mental Disorders. J. Nerv. Ment. Dis..

[9] Reynolds N., Mrug S., Wolfe K., Schwebel D., Wallander J. (2016). Spiritual coping, psychosocial adjustment, and physical health in youth with chronic illness: A meta-analytic review. Health Psychol. Rev..

[10] Shattuck E.C., Muehlenbein M.P. (2020). Religiosity/Spirituality and Physiological Markers of Health. J. Relig. Health.

[11] Sherman A.C., Merluzzi T.V., Pustejovsky J.E., Park C.L., George L., Fitchett G., Jim H.S.L., Munoz A.R., Danhauer S., Snyder M.A. (2015). A meta-analytic review of religious or spiritual involvement and social health among cancer patients. Cancer.

[12] Chen Y., Kim E.S., VanderWeele J. (2020). Religious-service attendance and subsequent health and well-being throughout adulthood: Evidence from three prospective cohorts. Int. J. Epidemiol..

[13] Parenteau S.C., Hurd K., Wu H., Feck C. (2019). Attachment to God and Psychological Adjustment: God’s Responses and Our Coping Strategies. J. Relig. Health.

[14] Pirutinsky S., Cherniak A.D., Rosmarin D.H. (2020). COVID-19, Mental Health, and Religious Coping among American Orthodox Jews. J. Relig. Health.

[15] Garduño-Ortega O., Morales-Cruz J., Hunter-Hernández M., Gany F., Costas-Muñiz R. (2021). Spiritual Well-Being, Depression, and Quality of Life among Latina Breast Cancer Survivors. J. Relig. Health.

[16] Hill P.C., Pargament K.I. (2003). Advances in the conceptualization and measurement of religion and spirituality. Implications for physical and mental health research. Am. Psychol..

[17] Austin P., Macdonald J., MacLeod R. (2018). Measuring Spirituality and Religiosity in Clinical Settings: A Scoping Review of Available Instruments. Religions.

[18] Das S., Punnoose V.P., Doval N., Nair V.Y. (2018). Spirituality, religiousness and coping in patients with schizophrenia: A cross sectional study in a tertiary care hospital. Psychiatry Res..

[19] Piccinini C.R.P., de Castro Almeida V., da Silva Ezequiel O., de Matos Fajardo E.F., Lucchetti A., Lucchetti G. (2021). Religiosity/Spirituality and Mental Health and Quality of Life of Early Pregnant Women. J. Relig. Health.

[20] Esan O., Lawal K. (2021). Spirituality and Suicidality among Cancer with Schizophrenia: A Cross-Sectional Study from Nigeria. J. Relig. Health.

[21] Wang X., Cheng Z. (2020). Cross-Sectional Studies: Strengths, Weaknesses, and Recommendations. Chest.

[22] Hackshaw A. (2008). Small studies: Strengths and limitations. Eur. Respir. J..

[23] Dilmaghani M. (2018). Importance of Religion or Spirituality and Mental Health in Canada. J. Relig. Health.

[24] Braam A.W., Koenig H.G. (2019). Religion, spirituality and depression in prospective studies: A systematic review. J. Affect. Disord..

[25] Park C.L., Lee S.Y. (2020). Unique effects of religiousness/spirituality and social support on mental and physical well-being in people living with congestive heart failure. J. Behav. Med..

[26] Koenig H.G. (2012). Religion, spirituality, and health: The research and clinical implications. ISRN Psychiatry.

[27] Mendes N.S., Malaguti C., Dos Anjos Sena L., Lucchetti G., De Jesús L., Vitorino L.M., Mesquita R., Lee A.L., Oliveira C.C. (2022). Spirituality and religiosity are associated with physical and psychological status in patients with chronic obstructive pulmonary disease. J. Clin. Nurs..

[28] Moons P., Luyckx K., Dezutter J., Kovacs A.H., Thomet C., Budts W., Enomoto J., Sluman M.A., Yang H.-L., Jackson J.L. (2019). Religion and spirituality as predictors of patient-reported outcomes in adults with congenital heart disease around the globe. Int. J. Cardiol..

[29] Li C., Hsieh C., Shih Y., Lin Y. (2021). Spiritual well-being of patients with chronic renal failure: A cross-sectional study. Nurs. Open.

[30] Li S., Stampfer M.J., Williams D.R., WanderWeele T.J. (2016). Association of Religious Service Attendance with Mortality among Women. JAMA Intern. Med..

[31] Moreira W.C., Nóbrega M., Lima F.P.S., Lago E.P., Lima M.O. (2020). Effects of the association between spirituality, religiosity and physical activity on health/mental health: A systematic review. Rev. Esc. Enferm. USP.

[32] Balboni T.A., VanderWeele T.J., Doan-Soares S.D., Long K.N.G., Ferrell B.R., Fitchett G., Koenig H.G., Bain P.A., Puchalski C., Steinhauser K.E. (2022). Spirituality in Serious Illness and Health. JAMA.

[33] Jones J.W. (2004). Religion, Health, and the Psychology of Religion: How the Research on Religion and Health Helps Us Understand Religion. J. Relig. Health.

[34] Aloush V., Gurevich-Shapiro A., Hazan E., Furer V., Elkayam O., Ablin J.N. (2021). Relationship between religiosity, spirituality and physical health and mental outcomes in fibromyalgia patients. Clin. Exp. Rheumatol..

[35] Park C.L., Sacco S.J., Kraus S.W., Mazure C.M., Hoff R.A. (2021). Influences of religiousness/spirituality on mental and physical health in OEF/OIF/OND military veterans varies by sex and race/ethnicity. J. Psychiatr. Res..

[36] Spanish Society of Medical Oncology (2022). Cancer Figures in Spain. https://seom.org/prensa/el-cancer-en-cifras.

[37] Koenig H.G. (2008). Concerns about measuring “spirituality” in research. J. Nerv. Ment. Dis..

[38] Głaz S. (2021). Psychological Analysis of Religiosity and Spirituality: Construction of the Scale of Abandonment by God (SAG). J. Relig. Health.

[39] Jim H.S.L., Pustejovsky J.E., Park C.L., Danhauer S.C., Sherman A.C., Fitchett G., Merluzzi T.V., Munoz A.R., George L., Snyder M.A. (2015). Religion, Spirituality, and Physical Health in Cancer Patients: A Meta-Analysis. Cancer.

[40] Park C.L., Sherman A.C., Jim H.S.L., Salsman J.M. (2015). Religion/Spirituality and Health in the Context of Cancer: Cross-Domain Integration, Unresolved Issues, and Future Directions: Religion/Spirituality and Health. Cancer.

[41] Page M.J., McKenzie J.E., Bossuyt P.M., Boutron I., Hoffmann T.C., Mulrow C.D., Shamseer L., Tetzlaff J.M., Akl E.A., Brennan S.E. (2021). PRISMA 2020 statement: An updated guide for the publication of systematic reviews. Rev. Esp. Cardiol..

[42] Law M., Stewart D., Letts L., Pollok N., Bosch J., Westmorland M. (1998). Guidelines for Critical Review Form—Quantitative Studies. https://healthsci.mcmaster.ca/docs/librariesprovider130/default-document-library/guidelines-for-critical-review-form-quantiative-studies-english.pdf?sfvrsn=ee9f6c19_2.

[43] Appelbaum M., Cooper H., Kline R.B., Mayo-Wilson E., Nezu A.M., Rao S.M. (2018). Journal Article Reporting Standards for Quantitative Research in Psychology: The APA Publications and Communications Board Task Force Report. Am. Psychol..

[44] Levitt H.M., Bamberg M., Creswell J.W., Frost D.M., Josselson R., Suárez-Orozco C. (2018). Journal Article Reporting Standards for Qualitative Primary, Qualitative Meta-Analytic, and Mixed Methods Research in Psychology: The APA Publications and Communications Board Task Force Report. Am. Psychol..

[45] Cannon A.J., Dokucu M.E., Loberiza F.R. (2022). Interplay between Spirituality and Religiosity on the Physical and Mental Well-Being of Cancer Survivors. Support. Care Cancer.

[46] Gielen J., Bhatnagar S., Chaturvedi S.K. (2017). Prevalence and Nature of Spiritual Distress among Palliative Care Patients in India. J. Relig. Health.

[47] Al-Natour A., Al Momani S.M., Qandil A.M.A. (2017). The Relationship between Spirituality and Quality of Life of Jordanian Women Diagnosed with Breast Cancer. J. Relig. Health.

[48] Bai M., Dixon J., Williams A., Jeon S., Lazenby M., McCorkle R. (2016). Exploring the Individual Patterns of Spiritual Well-Being in People Newly Diagnosed with Advanced Cancer: A Cluster Analysis. Qual. Life Res..

[49] Best A.L., Alcaraz K.I., McQueen A., Cooper D.L., Warren R.C., Stein K. (2015). Examining the Mediating Role of Cancer-Related Problems on Spirituality and Self-Rated Health among African American Cancer Survivors: A Report from the American Cancer Society’s Studies of Cancer Survivors-II: Spirituality, Cancer-Related Problems, and Self-Rated Health. Psycho-Oncology.

[50] Brown A.J., Sun C.C., Urbauer D., Zhukovsky D.S., Levenback C., Frumovitz M., Thaker P.H., Bodurka D.C., Ramondetta L.M. (2015). Targeting Those with Decreased Meaning and Peace: A Supportive Care Opportunity. Support. Care Cancer.

[51] Canada A.L., Murphy P.E., Fitchett G., Stein K. (2016). Re-Examining the Contributions of Faith, Meaning, and Peace to Quality of Life: A Report from the American Cancer Society’s Studies of Cancer Survivors-II (SCS-II). Ann. Behav. Med..

[52] Chaar E.A., Hallit S., Hajj A., Aaraj R., Kattan J., Jabbour H., Khabbaz L.R. (2018). Evaluating the Impact of Spirituality on the Quality of Life, Anxiety, and Depression among Patients with Cancer: An Observational Transversal Study. Support. Care Cancer.

[53] Cheng Q., Liu X., Li X., Wang Y., Mao T., Chen Y. (2019). Improving Spiritual Well-Being among Cancer Patients: Implications for Clinical Care. Support. Care Cancer.

[54] Goyal N.G., Ip E.H., Salsman J.M., Avis N.E. (2019). Spirituality and Physical Health Status: A Longitudinal Examination of Reciprocal Effects in Breast Cancer Survivors. Support. Care Cancer.

[55] Kamijo Y., Miyamura T. (2020). Spirituality and Associated Factors among Cancer Patients Undergoing Chemotherapy. Jpn. J. Nurs. Sci..

[56] Leeson L.A., Nelson A.M., Rathouz P.J., Juckett M.B., Coe C.L., Caes E.W., Costanzo E.S. (2015). Spirituality and the Recovery of Quality of Life following Hematopoietic Stem Cell Transplantation. Health Psychol..

[57] Sleight A.G., Boyd P., Klein W.M.P., Jensen R.E. (2021). Spiritual Peace and Life Meaning May Buffer the Effect of Anxiety on Physical Well-Being in Newly Diagnosed Cancer Survivors. Psycho-Oncology.

[58] Walker S.J., Chen Y., Paik K., Mirly B., Thomas C.R., Hung A.Y. (2017). The Relationships between Spiritual Well-Being, Quality of Life, and Psychological Factors before Radiotherapy for Prostate Cancer. J. Relig. Health.

[59] Yilmaz M., Cengiz H.Ö. (2020). The Relationship between Spiritual Well-Being and Quality of Life in Cancer Survivors. Palliat. Support. Care.

[60] Chen J., You H., Liu Y., Kong Q., Lei A., Guo X. (2021). Association between Spiritual Well-Being, Quality of Life, Anxiety and Depression in Patients with Gynaecological Cancer in China. Medicine.

[61] Rohde G.E., Young T., Winstanley J., Arraras J.I., Black K., Boyle F., Bredart A., Costantini A., Guo J., Irarrazaval M.E. (2019). Associations between Sex, Age and Spiritual Well-Being Scores on the EORTC QLQ-SWB32 for Patients Receiving Palliative Care for Cancer: A Further Analysis of Data from an International Validation Study. Eur. J. Cancer Care.

[62] Asgeirsdottir G.H., Sigurdardottir V., Gunnarsdottir S., Sigurbjörnsson E., Traustadottir R., Kelly E., Young T., Vivat B. (2017). Spiritual Well-Being and Quality of Life among Icelanders Receiving Palliative Care: Data from Icelandic Pilot-Testing of a Provisional Measure of Spiritual Well-Being from the European Organisation for Research and Treatment of Cancer. Eur. J. Cancer Care.

[63] Damen A., Exline J., Pargament K.I., Yao Y., Chochinov H., Emanuel L., Handzo G., Wilkie D.J., Fitchett G. (2021). Prevalence, Predictors and Correlates of Religious and Spiritual Struggles in Palliative Cancer Patients. J. Pain Symptom Manag..

[64] King S.D.W., Fitchett G., Murphy P.E., Pargament K.I., Martin P.J., Johnson R.H., Harrison D.A., Loggers E.T. (2017). Spiritual or Religious Struggle in Hematopoietic Cell Transplant Survivors: Spiritual or Religious Struggle in HCT Survivors. Psycho-Oncology.

[65] King S.D.W., Fitchett G., Murphy P.E., Rajaee G., Pargament K.I., Loggers E.T., Harrison D.A., Johnson R.H. (2018). Religious/Spiritual Struggle in Young Adult Hematopoietic Cell Transplant Survivors. J. Adolesc. Young Adult Oncol..

[66] Al Ahwal M.S., Al Zaben F., Sehlo M.G., Khalifa D.A., Koenig H.G. (2018). Religiosity and Telomere Length in Colorectal Cancer Patients in Saudi Arabia. J. Relig. Health.

[67] Hulett J.M., Armer J.M., Leary E., Stewart B.R., McDaniel R., Smith K., Millspaugh R., Millspaugh J. (2018). Religiousness, Spirituality, and Salivary Cortisol in Breast Cancer Survivorship: A Pilot Study. Cancer Nurs..

[68] Mendonça A.B., Pereira E.R., Magnago C., Costa Rosa Andrade Silva R.M., Meira K.C., de Oliveira Martins A. (2020). Distress and the Religious and Spiritual Coping of Brazilians Living with Cancer: A Cross-Sectional Study. Eur. J. Oncol. Nurs..

[69] Narayanan S., Milbury K., Wagner R., Cohen L. (2020). Religious Coping in Cancer: A Quantitative Analysis of Expressive Writing Samples from Patients with Renal Cell Carcinoma. J. Pain Symptom Manag..

[70] Pérez-Cruz P.E., Langer P., Carrasco C., Bonati P., Batic B., Tupper Satt L., Gonzalez Otaiza M. (2019). Spiritual Pain Is Associated with Decreased Quality of Life in Advanced Cancer Patients in Palliative Care: An Exploratory Study. J. Palliat. Med..

[71] Munoz A.R., Salsman J.M., Stein K.D., Cella D. (2015). Reference values of the Functional Assessment of Chronic Illness Therapy-Spiritual Well-Being: A report from the American Cancer Society’s studies of cancer survivors. Cancer.

[72] Leung C.H., Pong H.K. (2021). Cross-Sectional Study of the Relationship between the Spiritual Wellbeing and Psychological Health among University Students. PLoS ONE.

[73] Chirico F. (2016). Spiritual Well-Being in the 21st Century: It’s Time to Review the Current WHO’s Health Definition?. J. Health Soc. Sci..

[74] Peterman A.H., Fitchett G., Brady M.J., Hernandez L., Cella D. (2002). Measuring spiritual well-being in people with cancer: The functional assessment of chronic illness therapy—Spiritual Well-being Scale (FACIT-Sp). Ann. Behav. Med..

[75] Puchalski C.M. (2012). Spirituality in the cancer trajectory. Ann. Oncol..

[76] Cella D., Hernandez L., Bonomi A.E., Corona M., Vaquero M., Shiomoto G., Baez L. (1998). Spanish Language Translation and Initial Validation of the Functional Assessment of Cancer Therapy Quality-of-Life Instrument. Med. Care.

[77] Saiz J., Pung M.A., Wilson K.L., Pruitt C., Rutledge T., Redwine L., Taub P.R., Greenberg B.H., Mills P.J. (2020). Is Belonging to a Religious Organization Enough? Differences in Religious Affiliation versus Self-Ratings of Spirituality on Behavioral and Psychological Variables in Individuals with Heart Failure. Healthcare.

[78] Hvidt N.C., Nielsen K.T., Kørup A.K., Prinds C., Hansen D.G., Viftrup D.T., Hvidt E.A., Hammer E.R., Falkø E., Locher F. (2020). What is spiritual care? Professional perspectives on the concept of spiritual care identified through group concept making. BMJ Open.

[79] Büssing A. (2021). The Spiritual Needs Questionnaire in Research and Clinical Application: A Summary of Findings. J. Relig. Health.

[80] Toledo G., Ochoa C.Y., Farias A.J. (2021). Religion and Spirituality: Their Role in the Psychosocial Adjustment to Breast Cancer and Subsequent Symptom Management of Adjuvant Endocrine Therapy. Support. Care Cancer.

[81] Cowden R.G., Pargament K.I., Chen Z.J., Davis E.B., Lemke A.W., Glowiak K.J., Rueger S.Y., Worthington E.L. (2022). Religious/Spiritual Struggles and Psychological Distress: A Test of Three Models in a Longitudinal Study of Adults with Chronic Health Conditions. J. Clin. Psychol..

[82] Leavitt-Alcántara S., Betz J., Medeiros Almeida D., Ferrara B., Xu Y., Diop E., Hamilton O., Young C., Ragsdale J.R. (2022). Religiosity and Religious and Spiritual Struggle and Their Association to Depression and Anxiety among Adolescents Admitted to Inpatient Psychiatric Units. J. Health Care Chaplain..

[83] Rosmarin D.H., Pargament K.I., Mahoney A. (2009). The Role of Religiousness in Anxiety, Depression, and Happiness in a Jewish Community Sample: A Preliminary Investigation. Ment. Health Relig. Cult..

[84] Panier L.Y.X., Bruder G.E., Svob C., Wickramaratne P., Gameroff M.J., Weissman M.M., Tenke C.E., Kayser J. (2020). Predicting Depression Symptoms in Families at Risk for Depression: Interrelations of Posterior EEG Alpha and Religion Spirituality. J. Affect. Disord..

[85] Stripp T.K., Wehberg S., Büssing A., Andersen-Ranberg K., Jensen L.H., Henriksen F., Laursen C.B., Søndergaard J., Hvidt N.C. (2022). Protocol for EXICODE: The EXIstential health Cohort Denmark—A register and survey study of adult Danes. BMJ Open.

